# Oxidative Stress Induced Dysfunction of Protein Synthesis in 661W Mice Photoreceptor Cells

**DOI:** 10.3390/proteomes11020012

**Published:** 2023-04-03

**Authors:** Liting Deng, Vivek Gupta, Morteza Abyadeh, Nitin Chitranshi, Kanishka Pushpitha, Yunqi Wu, Veer Gupta, Yuyi You, Joao A. Paulo, Stuart L. Graham, Mehdi Mirzaei, Paul A. Haynes

**Affiliations:** 1 Macquarie Medical School, Faculty of Medicine, Health and Human Sciences, Macquarie University, Macquarie Park, NSW 2109, Australia; 2 ProGene Technologies Pty Ltd., Sydney, NSW 2113, Australia; 3 Australian Proteome Analysis Facility, Macquarie University, Macquarie Park, NSW 2109, Australia; 4 School of Medicine, Deakin University, Geelong, VIC 3220, Australia; 5 Department of Cell Biology, Harvard Medical School, Boston, MA 02115, USA; 6 School of Natural Sciences, Macquarie University, Macquarie Park, NSW 2109, Australia; 7 Biomolecular Discovery Research Centre, Macquarie University, Macquarie Park, NSW 2109, Australia

**Keywords:** proteomics, photoreceptor, oxidative stress, retina, ECM receptor interaction signalling pathway, oxidative phosphorylation, cellular senescence

## Abstract

Photoreceptor cells are highly susceptible to oxidative-stress-induced damage due to their high metabolic rate. Oxidative stress plays a key role in driving pathological events in several different ocular diseases, which lead to retinal degeneration and ultimately blindness. A growing number of studies have been performed to understand downstream events caused by ROS induced oxidative stress in photoreceptor cells; however, the underlying mechanisms of ROS toxicity are not fully understood. To shed light on ROS induced downstream pathological events, we employed a tandem mass tag (TMT) labelling-based quantitative mass-spectrometric approach to determine proteome changes in 661W photoreceptor cells following oxidative stress induction via the application of different concentrations of H_2_O_2_ at different time points. Overall, 5920 proteins were identified and quantified, and 450 differentially expressed proteins (DEPs) were identified, which were altered in a dose and time dependent manner in all treatment groups compared to the control group. These proteins were involved in several biological pathways, including spliceosome and ribosome response, activated glutathione metabolism, decreased ECM-receptor interaction, oxidative phosphorylation, abnormally regulated lysosome, apoptosis, and ribosome biogenesis. Our results highlighted ECM receptor interaction, oxidative phosphorylation and spliceosome pathways as the major targets of oxidative stress that might mediate vascular dysfunction and cellular senescence.

## 1. Introduction

Oxidative stress is defined as impaired balance between pro-oxidants and antioxidants, resulting in higher levels of reactive oxygen species (ROS) such as hydrogen peroxide (H_2_O_2_), nitric oxide (NO), superoxide (O_2_^−^) and highly reactive hydroxyl radicals (HO). It plays a key role in the initiation and progression of several retinal degenerative diseases such as diabetic retinopathy (DR), glaucoma and age-related macular degeneration (AMD) [1,2]. Oxidative stress and accompanying neurodegeneration are also involved in several neurodegenerative disorders including Alzheimer’s disease (AD), Parkinson’s disease (PD) and Huntington’s disease (HD) [3,4]. Photoreceptor cells account for about 60% of all cells in the retina; these neurons are also the most metabolically active neurons in the central nervous system, containing around 75% of all retinal mitochondria which are a primary source of ROS generation [5,6]. This feature, along with continuous exposure of the eye to environmental stimuli such as varying light intensities, UV radiation, smoke, air pollution and oxygen, makes these cells vulnerable to oxidative stress insults. Cumulative evidence suggests that oxidative stress contributes to DNA, lipid and protein damage and, consequently, cell death. However, the underlying mechanisms of these pathological events following oxidative stress in photoreceptor cells remain ill-defined [7,8]. 

The accumulation of β-amyloid (Aβ) in the brain, known as the amyloid plaque, is a key pathological hallmark of AD that is also reported in the retina of glaucoma, DR and AMD subjects, suggesting overlapping mechanisms between AD and these conditions. Interestingly, Aβ has been suggested as a trigger of oxidative stress in these diseases [9,10]. In this regard, a previous proteomics study by our group showed increased oxidative stress in retinal photoreceptor cells upon treatment with Aβ, and investigated the underlying mechanisms of Aβ toxicity on photoreceptor cells [11]. However, increased oxidative stress has also been observed in the absence of Aβ, and it has also been suggested that it may promote the aggregation and accumulation of the peptide [12,13,14,15,16]. Therefore, exploring the oxidative stress induced changes independent of Aβ accumulation can provide unique insights into oxidative-stress-derived pathological events. 

Herein, we studied the proteome changes in photoreceptor cells in oxidative stress conditions induced by different concentrations of H_2_O_2_ in different times of exposure, using tandem mass tag (TMT) quantitative proteomics. We also compared the identified differentially expressed proteins (DEPs) with previously reported DEPs in photoreceptor cells upon treatment with Aβ, to reveal the common and distinct altered pathways.

## 2. Materials and Methods

### 2.1. Cell Culture and Treatments

Mouse photoreceptor-derived 661W cells were kindly provided by Prof. Al-Ubaidi, University of Oklahoma. The cells were cultured in DMEM medium containing 10% (*v/v*) fetal bovine serum (FBS), 1% (*v/v*) penicillin/streptomycin (Thermo Fisher, San Jose, CA, USA). Cultures were maintained at 37 °C under a humidified atmosphere of 5% CO_2_, as previously described [11,17]. The cells were exposed to two different concentrations of H_2_O_2_ (Sigma, St Louis, MO, USA),10 µM and 50 µM, and then samples were separately collected at different times of exposure; after 0.5 h in treatments with 10 µM H_2_O_2_, and after 6 h and 12 h in treatments with 50 µM H_2_O_2_. Therefore, there were four distinct sampling points including control (CTRL), concentration 1 (C1) (10 µM, 0.5 h), concentration 2 (C2) (50 µM, 6 h), and concentration 3 (C3) (50 µM, 12 h). Three biological replicates were prepared for each specific condition. Cells were stored at −20 ℃ until protein extraction.

### 2.2. Protein Sample Preparation

Protein samples were prepared as described previously [11]. Briefly, cells were solubilized in cold lysis buffer (20 mM HEPES, pH 7.4, 150 mM NaCl, 1% Triton x-100, 1 mM EDTA) containing protease inhibitor under sonication. Lysates were centrifuged for 15 min at 13,000 rpm at 4 ℃ and supernatant was collected, reduced with dithiothreitol (20 mM) for 30 min, and alkylated with 50 mM iodoacetamide for 30 min in the dark at room temperature. An additional 40 mM DTT was added for 15 min to quench the alkylation reaction. Protein precipitation and contaminant removal were performed using chloroform–methanol extraction [18]. Samples were air-dried for evaporation of the remaining methanol and then resuspended in 650 μL HEPES buffer (pH 8.8). Total protein quantities were measured using a BCA Protein Assay kit (Pierce, Rockford, IL, USA). An aliquot of 150 μg of proteins was digested with trypsin (protein: enzyme 50:1) at 37 °C overnight. Digestion was stopped by placing samples on ice and adjusting to a final concentration of 1 mM trifluoroacetic acid, followed by measurement of the peptide concentration using a Micro BCA assay kit (Pierce, Rockford, IL, USA). 

### 2.3. TMT Labelling, LC-MS/MS Analysis and Peptide to Spectrum Matching

An aliquot of 60 μg peptides from each sample was labelled with 0.25 mg of TMT reagent (Thermo Fisher, San Jose, CA, USA) at room temperature for one hour with occasional vortexing. To quench the reaction, 8 μL of 5% fresh hydroxylamine was added to each sample, and then it was incubated at room temperature for 15 min with additional vortexing. TMT labelling was performed using three separate 10-plex labelling kits, as part of a larger scale experiment, so only the first four channels of labelling were used in each set. For the first set of TMT labels, designated R1, the labels were used as follows: control R1 = 126 C, (C1) (10 µM, 0.5 h) R1 = 127 N, (C2) (50 µM, 6 h) R1 = 127 C, and (C3) (50 µM, 12 h) R1 = 128 N. For the second set of TMT labels, designated R2, the labels were used as follows: control R2 = 126 C, (C1) (10 µM, 0.5 h) R2 = 127 N, (C2) (50 µM, 6 h) R2 = 127 C, and (C3) (50 µM, 12 h) R2 = 128 N. For the third set of TMT labels, designated R3, the labels were used as follows: control R3 = 126 C, (C1) (10 µM, 0.5 h) R3 = 127 N, (C2) (50 µM, 6 h) R3 = 127 C, and (C3) (50 µM, 12 h) R3 = 128 N. 

Following the labelling reaction, peptide samples were combined and evaporated to dryness in a vacuum centrifuge. The TMT labelled peptide mixture was redissolved in 1% formic acid and a Sep-Pak C18 cartridge (Waters, Milford, MA, USA) was employed to de-salt the mixture. TMT labelled peptides were then separated using a high pH reversed-phase peptide fractionation kit (Thermo Fisher, San Jose, CA, USA), with a total of 17 peptide containing fractions collected. These were evaporated to dryness, and peptides were redissolved in 1% formic acid and desalted for a second time using SDB-RPS (3M-Empore) Stage Tips. A nano flow liquid chromatography—tandem mass spectrometry system consisting of an EASY-nLC1000 nanoflow HPLC system (Thermo Scientific, San Jose, CA, USA) coupled to a Q Exactive Orbitrap mass spectrometer (Thermo Scientific, San Jose, CA, USA) was employed for the acquisition of mass spectrometric data for the identification and quantification of peptides.

Peptide to spectrum matching was performed using Sequest HT and Mascot algorithms in Proteome Discoverer V2.1 (Thermo Scientific, San Jose, CA, USA). Spectra were searched against the SwissProt *Mus Musculus* protein sequence database (http://www.ebi.ac.uk/swissprot/ (16,953 sequences, accessed on 16 February 2019)), with parameters as described previously [11,19,20]. Parent ion MS tolerance was specified as ±10 ppm, while fragment ion MS/MS tolerance was specified as 0.02 Da. Carbamidomethylation of cysteine was considered to be a fixed modification. A range of variable modifications were permitted, including Deamidation (N, Q), Acetylation (Protein N-Terminus), Glu- > pyro-Glu (N-term E), Gln- > pyro-Glu (N-term Q), Oxidation (M), and TMT 10-plex labelling (N-term, K). The discrimination of peptide to spectrum matches, which involved calculating statistics including posterior error probabilities and Q-values, was achieved using the Percolator algorithm. Further filtering was performed to retain only peptides and proteins with FDR less than 1%. A summary of all identify proteins is included in Supplementary Table S1, while complete details of all identify proteins are included in Supplementary Table S2, including Sequest HT and Mascot Scores, peptide sequences and modifications, and details of which high pH reversed-phase peptide fractions the labelled peptides were identified in.

### 2.4. Analysis of Differentially Expressed Proteins

TMTPrepPro [21] was used for further quantitative analysis of identified proteins. The control reference (label 126) was used for the calculation of protein ratios, which were extracted from individual experiments and combined across all the runs involved. Differentially expressed proteins (DEPs) were identified across all conditions using two-way analysis of variance (ANOVA). Additional comparisons were performed using pairwise analysis of each treatment relative to control using the Student’s t-test. Abundance ratios of DEPS identified in ANOVA comparisons were log transformed prior to clustering. In comparisons performed in a pairwise manner, the relative quantitation of protein after peroxide treatment of cells was performed using the ratio of the detected TMT label in each of the treatments compared to the matched control. Geometric means of the respective ratios were used to calculate overall fold changes for individual proteins.

Proteins were considered to be DEPs if they met the following criteria: fold change was greater than 1.20 (increased) or less than 0.833 (decreased), with *p*-value less than 0.05 [22,23]. Finally, the identified DEPs were subjected to further bioinformatics analyses, including Gene Ontology (GO) and pathway enrichment analysis using the online tool String [24,25]. 

## 3. Results

### 3.1. Proteome Profiling of Photoreceptor Cells and Significantly Regulated Proteins by H_2_O_2_ Treatments

**Figure 1:**
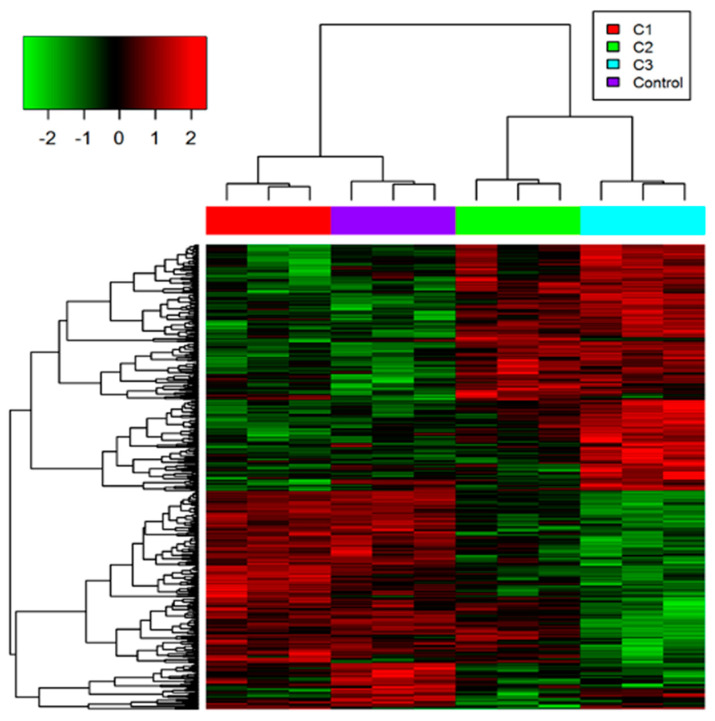
Heatmap (hierarchical clustering) of the log-transformed ratios of differentially expressed proteins (differences between all experimental conditions via analysis of variance (ANOVA)) after H_2_O_2_ treatments, row clustering only. Column colours indicate treatment types.

A total of 5920 proteins was identified from 661W photoreceptor cells in this study (Supplementary Table S1). These proteins were quantified with multiple peptides at an initial protein FDR less than 1%. There were 410 proteins identified as differentially abundant under different conditions based on the ANOVA analysis (which met the requirements of both *p*-value < 0.05 and the absolute value of fold change > 1.2). Hierarchical clustering analysis was applied to those differentially expressed proteins and showed similar expression patterns of proteins increased or decreased in abundance between 6 h and 12 h of 50 µM H_2_O_2_ treatments (C2 and C3, respectively) (Figure 1). However, fewer proteins were changed in abundance after 0.5 h of 10 µM H_2_O_2_ treatment (C1), indicating that the cellular response under this relatively mild condition was quite distinct to that observed for the other two H_2_O_2_ treatments. 

**Figure 2:**
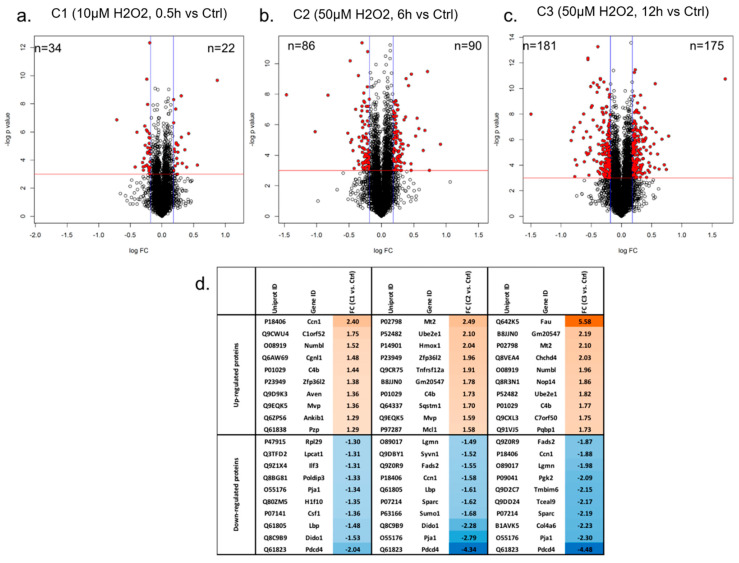
(**a**–**c**) Volcano plots demonstrating the dual thresholds for differentially regulated proteins in photoreceptor cells under three different H_2_O_2_ treatments: C1 (10 µM, 0.5 h), C2 (50 µM, 6 h), and C3 (50 µM, 12 h). Each data point represents a single quantified protein. The x-axis represents log fold change in abundance (H_2_O_2_ treatment vs. control). Vertical blue lines indicate ratios 1.2 and 0.833. The −log (*p*-value) is plotted on the y-axis. Proteins above the red horizontal line indicate significance lower than 0.05. Proteins within the upper and outer quadrants meet both the fold change and *p*-value cut-off, and are therefore considered to be differentially abundant. (**d**) Top 10 differential proteins in cells under three different H_2_O_2_ treatments, sorted by fold change values. Proteins increased in abundance by H_2_O_2_ treatment are highlighted in orange, with colour intensity corresponding to fold change values, while proteins decreased in abundance by H_2_O_2_ treatment are highlighted in blue, with colour intensity corresponding to fold change values.

The pairwise analysis between the mildest H_2_O_2_ treatment (10 µM, 0.5 h) versus control yielded 22 increased (*p*-value < 0.05 and fold change > 1.2) and 34 decreased proteins (*p*-value < 0.05 and fold change < −1.2), respectively (Figure 2a and Supplementary Table S3). In comparison, a greater portion of proteins was differentially expressed when cells were treated with a higher concentration of H_2_O_2_ (50 µM). There were 90 proteins increased, and 86 proteins decreased in abundance, after cells were exposed to 50 µM H_2_O_2_ for 6 h (Figure 2b). More proteins responded to H_2_O_2_ when cells were treated for a longer duration (12 h) at this concentration (50 µM), with 175 and 181 proteins being increased and decreased in abundance, respectively (Figure 2c). 

The top 10 differentially expressed proteins according to fold change values in photoreceptor cells under three specific H_2_O_2_ treatments were further analysed (Figure 2d). The fold changes of the differentially abundant proteins ranged from 2.4 to −2.04 under C1 (10 µM, 0.5 h), 2.49 to −4.34 under C2 (50 µM, 6 h), and 5.58 to −4.48 under C3 (50 µM, 12 h). This indicates that impacts of H_2_O_2_-induced oxidative stress on photoreceptor cells increased when they were exposed to a higher concentration of H_2_O_2_ and treated for a longer time. Among these, four proteins were differentially abundant in all three conditions, including Ccn1, C4b, Pja1, and Pdcd4. Ccn1, a 40-kDa heparin-binding protein rich in cysteine residues and known as a connective tissue growth factor, was increased in abundance in C1 (10 µM, 0.5 h) where a lower concentration of H_2_O_2_ and a shorter time were applied to cells.

However, the abundance of Ccn1 significantly decreased when cells were treated with 50 µM H_2_O_2_ for either 6 or 12 h. C4 binding protein (C4b), which is essential for the propagation of the classical complement pathway, showed increased expression in cells under all these three treatment conditions. Pja1 protein is one of the RING-finger E3 ligases that are instrumental in the regulation of inflammatory cascades, apoptosis, and cancer. A reduced abundance of this protein was found in cells after all three H_2_O_2_ treatments. Similarly, the abundance of programmed cell death protein 4 (Pdcd4) also decreased in all conditions of oxidative stress. Pdcd4 showed the greatest decrease in abundance in response to all H_2_O_2_ treatments (average fold change of −3.62), while 40S ribosomal protein S30 (FAU) showed the greatest increase in abundance (5.58-fold change), in C3. 

**Figure 3:**
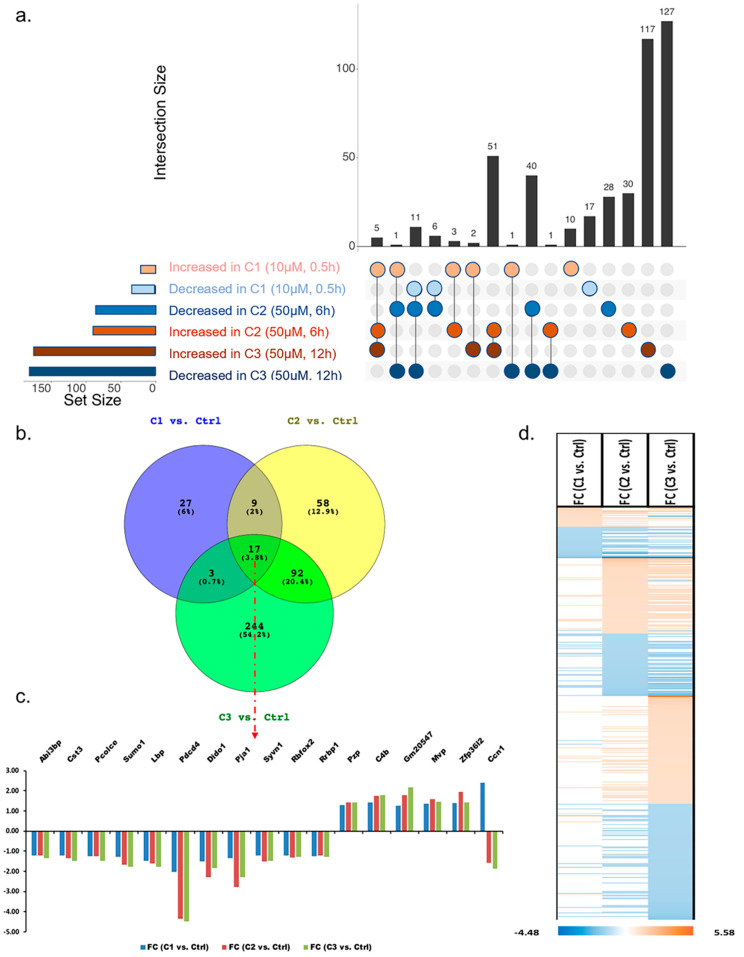
(**a**) Upset plot showing the overlap of proteins with either increased or decreased abundance in three different treatments. Blue and orange colours separately represent the decreased and increased abundance of proteins in photoreceptor cells under H_2_O_2_ treatment. (**b**) Distribution of differentially abundant proteins relative to control in C1, C2 and C3. (**c**) Seventeen common altered proteins and their expression changes in C1, C2, and C3. (**d**) Heatmap indicating the expression pattern of 450 proteins with significant changes in abundance in photoreceptors under specific treatments. Orange and blue colours represent an increase in abundance (t-test, FC > 1.2, *p*-value < 0.05) and decrease in abundance (*t*-test, FC < −1.2, *p*-value < 0.05), respectively.

A total of 450 proteins were differentially abundant in response to the three treatments when compared with the controls using pairwise analysis. Similar yet different changes in protein abundance were found among three treatments with changes to applied concentrations of H_2_O_2_ and exposure time (Figure 3a,b). Changes in protein abundance generally increased with the more concentrated H_2_O_2_ and treatment time. The common differentially abundant proteins between any two treatments were 26 proteins between C1 and C2, 20 proteins between C1 and C3, and 109 proteins between C2 and C3, respectively. The expression patterns of most common proteins in two treatments were the same with either increase or decrease in abundance (Figure 3c,d). A total of 17 proteins were commonly identified as differentially expressed in all three H_2_O_2_ treatments, with 11 of these 17 proteins decreased in abundance, while five were increased in abundance. Ccn1 was the only protein with decreased abundance in C1 (10 µM, 0.5 h), but significantly increased abundance in C2 (50 µM, 6 h) and C3 (50 µM, 12 h), as mentioned above. All of these 17 proteins showed very similar expression patterns between C2 and C3 when a higher concentration and longer treatment time were applied to photoreceptor cells. 

#### 3.1.1. GO Analysis of Regulated Proteins 

**Figure 4:**
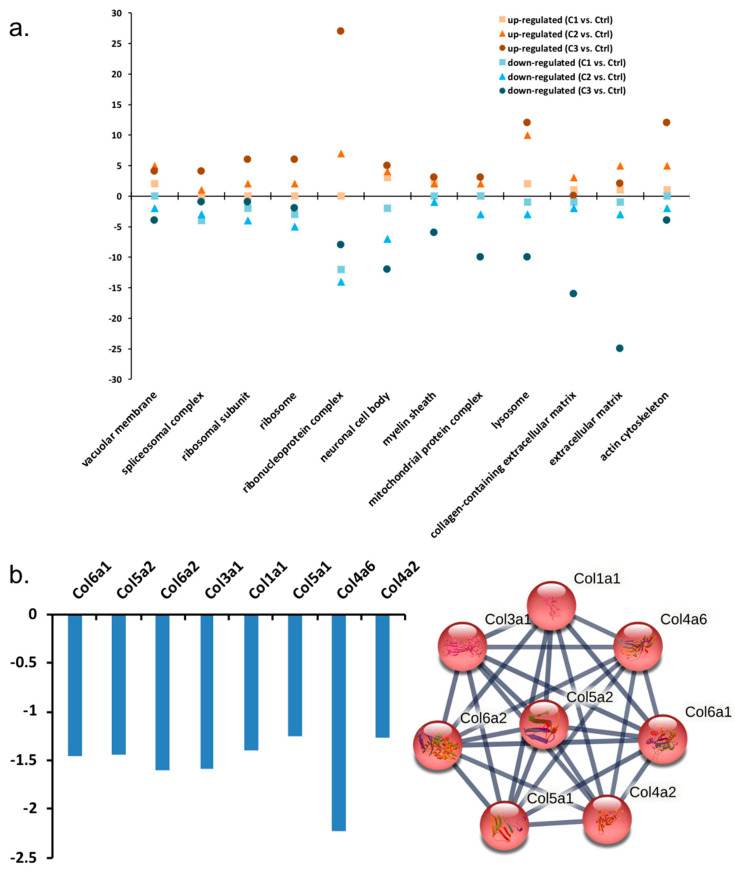
(**a**) GO analysis showing the number of proteins increased in abundance (orange colour) and decreased in abundance (blue colour), classified into the specific cellular component, biological pathway, and molecular function in photoreceptor cells under H_2_O_2_ treatments. Different shapes of markers indicate specific treatment (square—C1, triangle—C2, round—C3). (**b**) Significantly decreased collagen proteins in C3 (50 µM, 12 h) by H_2_O_2_ (fold change < −1.2, *p*-value < 0.05) and their interactions.

Gene ontology analysis indicated the response of proteins that were classified to a specific cellular component, biological process, and molecular function (Figure 4a). More than 25 proteins belonging to the ribonucleoprotein complex responded with increased abundance to the H_2_O_2_ treatment after 12 h. However, more than 25 proteins involved in regulating various aspects of the extracellular matrix, including eight collagen proteins, were decreased in abundance under the same conditions (Figure 4a,b). The number of responsive proteins in different groups mostly increased with the increasing concentration of H_2_O_2_ treatment, such as components from ribosome, lysosome, and actin cytoskeleton (Figure 4a). 

#### 3.1.2. Pathway Classification of Differentially Abundant Proteins

**Figure 5:**
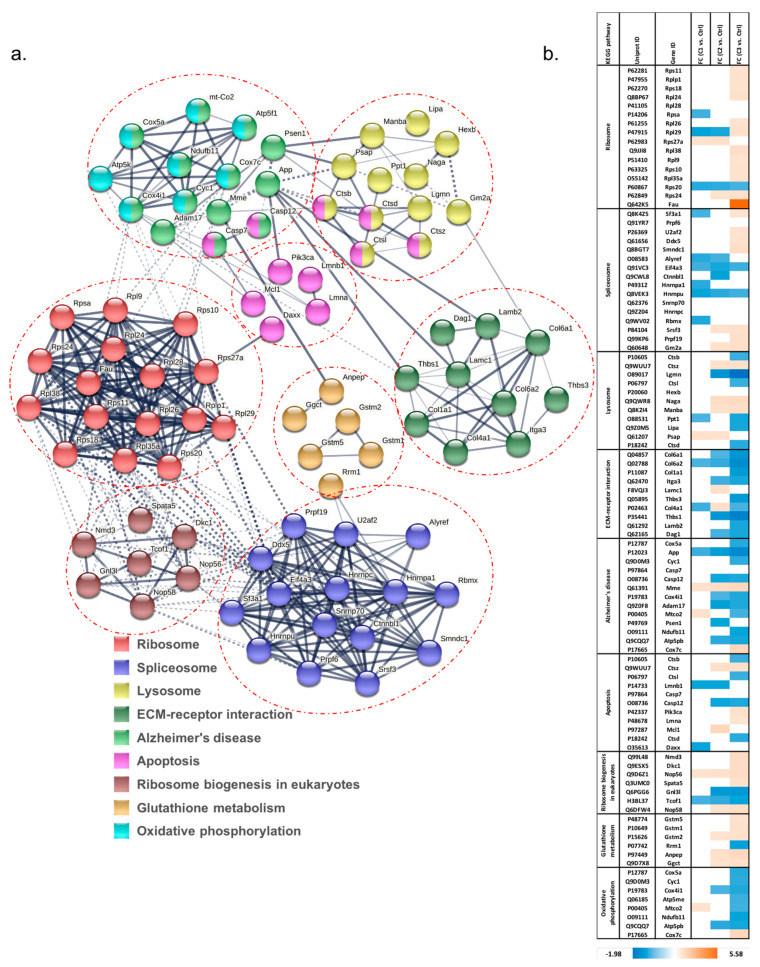
(**a**) Network of protein interactions and regulated KEGG pathways by H_2_O_2_ treatment. (**b**) The change in abundance of proteins demonstrating the response of specific KEGG pathways, including increased spliceosome and ribosome response, activated glutathione metabolism, decreased ECM-receptor interaction and oxidative phosphorylation, abnormally regulated lysosome, apoptosis, and ribosome biogenesis.

The network of interacting proteins that were differentially expressed among all groups based on ANOVA was generated using String. KEGG pathway analysis was used to display the altered pathways, including ribosome, spliceosome, lysosome, ECM-receptor interaction, Alzheimer’s disease, apoptosis, ribosome biogenesis in eukaryotes, glutathione metabolism, and oxidative phosphorylation (Figure 5a,b). As expected, C3 (50 µM, 12 h) treatment produced the maximum number of molecules regulated in these KEGG pathways. Most ribosome and spliceosome proteins were increased in abundance. In addition, three glutathione S-transferases—Gstm1, Gstm2 and Gstm5—were found with increased abundance under C3, indicating activated glutathione metabolism. In addition, proteins involved in extracellular matrix (ECM)-receptor interaction were decreased in abundance. Oxidative phosphorylation was disrupted, with seven out of eight proteins in this pathway decreased in abundance by longer and more concentrated H_2_O_2_ treatment. Amyloid precursor protein (APP), belonging to the Alzheimer’s pathway, was decreased in abundance in cells with increased H_2_O_2_ treatments, and had fold changes of −1.14, −1.35, and −1.63 in C1, C2, and C3, respectively (all *p*-value < 0.05). Another protein in this pathway, presenilin 1 (Psen1), was also decreased in abundance under C2. Intriguingly, membrane metallo-endopeptidase (MME), which is considered one of the important β-amyloid (Aβ)-degrading enzymes related to prevention of Alzheimer’s disease (AD) pathology [26], was identified with increased abundance in all H_2_O_2_ treatment groups. This may occur as part of an induced compensatory response against H_2_O_2_ toxicity, which in turn decreased levels of target proteins including β-amyloid [27]. Additionally, the increased abnormal regulation of proteins in KEGG pathways related to lysosomes, apoptosis, and ribosome biogenesis was identified with more severe H_2_O_2_ treatment as well.

#### 3.1.3. Analysis of Proteins Found to Be Differentially Abundant in 661W Photoreceptor Cells by Both H_2_O_2_ and Aβ Treatments

The results of this study were compared with those of our previously published study, in which the same cell line was treated with different concentrations of Aβ peptides. A total of 4636 out of 5837 proteins identified in photoreceptor cells under Aβ treatments were also found in this study. Among those, 61 proteins were changed in abundance in 661W photoreceptor cells by both H_2_O_2_ and Aβ treatments with either different concentrations or treatment time-points (Supplementary Figure S1). Similar yet distinct changes in abundance and proteome complexity were identified between these two studies on photoreceptor cells, which separately mimicked the impact of Alzheimer’s disease and oxidative stress on the eye. For example, the Aβ degrading enzyme and membrane protein Mme was increased in abundance in both studies when cells were treated with either H_2_O_2_ induced oxidative stress or longer Aβ treatment (12 h), which was believed to cause more severe oxidative stress-mediated toxic effects. However, cathepsin D (Ctsd), which is a lysosomal acid protease and plays a role in APP processing, the clearance of Aβ, and intracellular protein breakdown, was decreased in abundance in cells under the more severe Aβ (25 µm, 12 h) and H_2_O_2_ (50 µM, 12 h) treatments. Further GO analysis classified 34 out of these 61 proteins into specific groups, including different biological processes, molecular functions, or cellular components (Supplementary Figure S2). This analysis identified six and nine components in the biological process of ribosome biogenesis and RNA processing, as well as 11 proteins with RNA binding molecular functions. In addition, the cellular component analysis indicated that five, six, and eight differentially abundant proteins belonged to the actin cytoskeleton, ribosomal subunit, and lysosomal networks, respectively.

## 4. Discussion

Photoreceptor cells, including rods and cones, are retinal neuroepithelial cells that are crucial for visual phototransduction. Oxidative stress is now well established as a major player in photoreceptor cell degeneration; however, the downstream changes are not well defined [28,29]. We performed this study to investigate downstream events at the protein level in a retinal cone cell line (661W) following exposure to different concentrations of an oxidative stress inducer at different time points.

Results of the present study showed that H_2_O_2_ exposure altered the proteome complexity of 661W photoreceptor cells in a time and concentration dependent manner. Overall, 450 DEPs were identified in all treatment groups and several significantly altered biological pathways were identified, including ribosome, spliceosome, ECM-receptor interaction, oxidative phosphorylation, and glutathione metabolism. Moreover, 17 of these 450 DEPs were common between all three treatment groups, which were mainly involved in protein synthesis. 

The alteration of ribosomal proteins in retinal disorders has been reported previously [30,31]. In C1 treatment, a small number of ribosomal proteins were decreased in abundance while a greater proportion of proteins was increased in abundance when exposed to higher concentration of H_2_O_2_ for a longer time. In agreement with our results, the down-regulation of several ribosomal genes was observed in a rat model of chronic glaucoma with early nerve injury, while more ribosomal genes were found to be up-regulated with advanced nerve injury [32]. The increased expression of ribosomal genes has also been observed in retinal pigment epithelium (RPE)-choroid-sclera samples of AMD patients and aqueous humour (AH) of primary open-angle glaucoma (POAG) patients [29]. Ribosomes are very important in protein synthesis, so a decrease in the abundance of some ribosomal proteins at lower concentrations of H_2_O_2_ is possibly due to initial induced stress, while exposure to higher concentrations for longer time resulted in a considerable number of proteins increasing in abundance, which might be due to the increased production of proteins involved in spliceosome, glutathione metabolism and apoptosis, or might be a compensatory response for cell survival [33]. In addition, a decrease in the abundance of Pdcd4 (which inhibits translation initiation) in all treatment groups also correlates with increased protein production [34]. 

The alteration of RNA splicing has been previously reported in human samples of POAG and AMD by our group and others [35,36,37]. As shown in Figure 5b, treatment with 10 µM H_2_O_2_ for 0.5 h resulted in a decreased abundance of several spliceosome proteins which were then shifted towards an increase in abundance following exposure to higher concentrations of H_2_O_2_ (50 µM, 6 h), and which increased even more upon a longer time of exposure (50 µM, 12 h). This change in abundance of the spliceosome proteins, particularly U2af2 and Prpf19, might be a compensatory response reflecting the initiation of global spliceosome activity in the early stage of cellular senescence, which is triggered by H_2_O_2_ induced oxidative stress [38,39]. 

The ECM consists of collagens, proteoglycans and glycoproteins that support the maintenance of cell structure and provide a scaffold essential for maintaining the organization of vascular endothelial cells into blood vessels. Interactions between ECM and cells are crucial for cell adhesion, migration, differentiation, proliferation, and apoptosis [40,41]. We observed a significant decrease in the abundance of collagen and laminin proteins, including col5a1/2, col6a1/2, col4a2/6, col1a1 col3a1, lamb2, and lamc1, especially in C3 treatment. The down-regulation of genes involved in collagen synthesis and extracellular matrix organization has been reported in both human samples and cell models of keratoconus (KTCN), another eye disorder [42,43]. Laminin and collagen are involved in vasculature and blood vessel development [44,45]. The outer retina, containing photoreceptor cells, is known to be the most metabolically active tissue in the body, and the choroidal and retinal vasculature is developed to meet the specialized retina metabolic requirements. The retina has two vascular systems, including a central retinal artery in the inner retina and the choriocapillaris in the retinal pigment epithelium and outer retina [46]. Choroidal vascular dysfunction and oxidative stress are two well-established factors in AMD pathogenesis [47,48], but little is currently known about the interplay between these two pathophysiological factors. Herein, our results showed that H_2_O_2_-induced oxidative stress resulted in a decreased abundance of collagen and laminin proteins. It is of interest that the decreased expression of ECM components, particularly collagen genes including COL1A1, COL1A2, and COL3A1, has also been reported in retinal pigment epithelial cells upon cellular senescence [49,50]. Moreover, the down-regulation of collagen genes such as COL3A1, COL4A1, COL4A2, and COL5A1 has been reported following cellular senescence induction in hepatic stellate cells and COL16A1 in human dermal fibroblast cells [51,52]. 

Mitochondrial dysfunction, and consequently impaired oxidative phosphorylation, is another well-known feature of cellular senescence [53] and has also been observed in this study. Oxidative phosphorylation is an important metabolic pathway and a major source of energy that occurs in the mitochondria. Oxidative phosphorylation impairment due to mitochondrial dysfunction has been widely reported in many eye diseases such as DR, glaucoma and AMD [54,55,56,57]. Therefore, mitochondrial dysfunction has become a well-established part of these disorders that drive disease progression. Photoreceptor cells require large amounts of ATP, and therefore healthy mitochondria are crucial for cellular survival and function, and impaired mitochondrial function leads to photoreceptor degeneration [58,59]. 

Finally, another biochemical module that was enriched was glutathione metabolism, which remained relatively unchanged in C1 (10 µM, 6 h) but was significantly increased in abundance in C2 (50 µM, 6 h) and further increased with increasing time of exposure in C3 (50 µM, 12 h). Glutathione is the most abundant antioxidant molecule within the cell, and counteracts the effects of ROS and H_2_O_2_ to prevent cellular damage. Although glutathione has been reported to be decreased in DR, AMD and glaucoma [60], it was increased in abundance in our study, which has also been reported in early-stage AMD patients. This is possibly due to an initial compensatory response to neutralize the increased H_2_O_2_-induced oxidative stress and prevent cell senescence [61]. 

Interestingly, comparing these data with our previous findings in the same photoreceptor cell line upon treatment with Aβ [11] yielded 61 DEPs that were common between all treated groups in the two studies (Supplementary Figure S1). These proteins were involved in ribosome biogenesis, ribosomal subunits, lysosome, actin cytoskeleton, and RNA binding and processing (Supplementary Figure S2). In addition to these pathways, oxidative phosphorylation was also enriched in both studies. Intriguingly, these alterations were observed in decreased levels of β-amyloid, and therefore our results support the key role of oxidative stress in neurodegenerative diseases that may precede β-amyloid deposition [62].

## 5. Conclusions

The results of the present study highlight the biological pathways affected by oxidative stress in photoreceptor cells, and provide a set of differentially abundant proteins which are a valuable resource for future animal and human cell studies focused on oxidative stress response. In addition, our findings suggest the impairment of ECM-receptor interaction, oxidative phosphorylation, and spliceosome as the major targets of H_2_O_2_-induced oxidative stress, which might contribute to vascular dysfunction and photoreceptor cell degeneration. 

## 6. Limitations

This study has some inherent limitations due to the fact that it is a bottom-up proteomics analysis which involves first breaking proteins down into peptides. We acknowledge that this means this manuscript contains little or no information regarding different proteoforms, because that information is mostly lost when protease digestion is employed in the first stage of sample preparation. There is also little information in this manuscript on protein species, because we did not attempt to address any particular posttranslational modifications; rather, the study was focused on individual gene products. 

Proteomes are, by their very nature, complex systems. Describing the components of a complex system in terms of what is there and how much of it is present under different conditions is an essential first step in understanding how a complex system works. Comparative quantitative proteomics studies such as the one presented here are essential building blocks in understanding the overall proteome complexity involved in different cells and how they respond to external stress. Despite these caveats, identifying and quantifying proteins changed in abundance in photoreceptor cells’ responses to hydrogen peroxide demonstrates the power of proteomic analysis in the discovery of biomarkers of oxidative stress, and provides an important foundation for further investigation.

## Data Availability

Mass spectrometric data are available via ProteomeXchange with the identifier PXD037329.

## Supplementary Materials

The following supporting information can be downloaded at: https://www.mdpi.com/article/10.3390/proteomes11020012/s1. Supplementary Table S1—Details of all 5920 proteins identified from 661W photoreceptor cells in this study; Supplementary Table S2—Complete details of identified proteins identified in this study including peptide sequences, and Sequest HT and Mascot Scores; Supplementary Table S3—Differentially abundant proteins identified in all three treatments; Supplementary Figure S1—Heat map table of differentially expressed proteins in 661W cells treated by H_2_O_2_ and Aβ when compared to the control; Supplementary Figure S2—Network analysis of 61 commonly regulated proteins by both H_2_O_2_ and Aß in 661 photoreceptor cells.
