# Supplementary material for: Oxidative Stress Induced Dysfunction of Protein Synthesis in 661W Mice Photoreceptor Cells
Source: Proteomes. 2023 Apr 3;11(2):12. doi: 10.3390/proteomes11020012 (PMC10123756; doi:10.3390/proteomes11020012)
Supplement: Supplementary file 1 [file proteomes-11-00012-s001.zip › Deng et al Supplementary Figures 4dec22.pdf]

Supplementary Figures for Deng et al, 4 Dec 2022

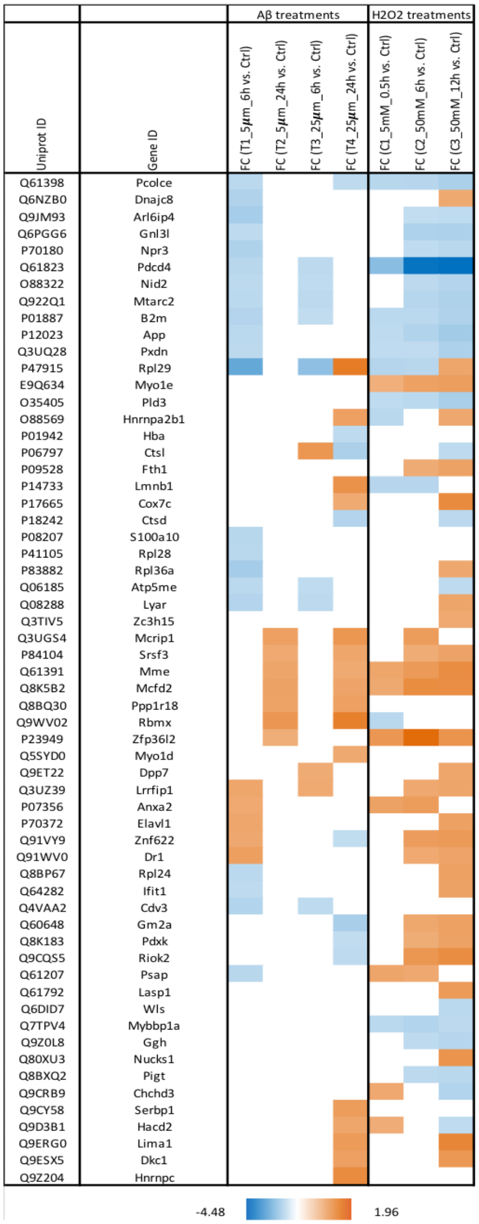

Supplementary Figure S1. Heat map table of differentially expressed proteins in 661W cells treated by H_2O_2 and Aβ when compared to the control. Orange and blue bars represent the proteins increased and decreased in abundance in specific treatments.

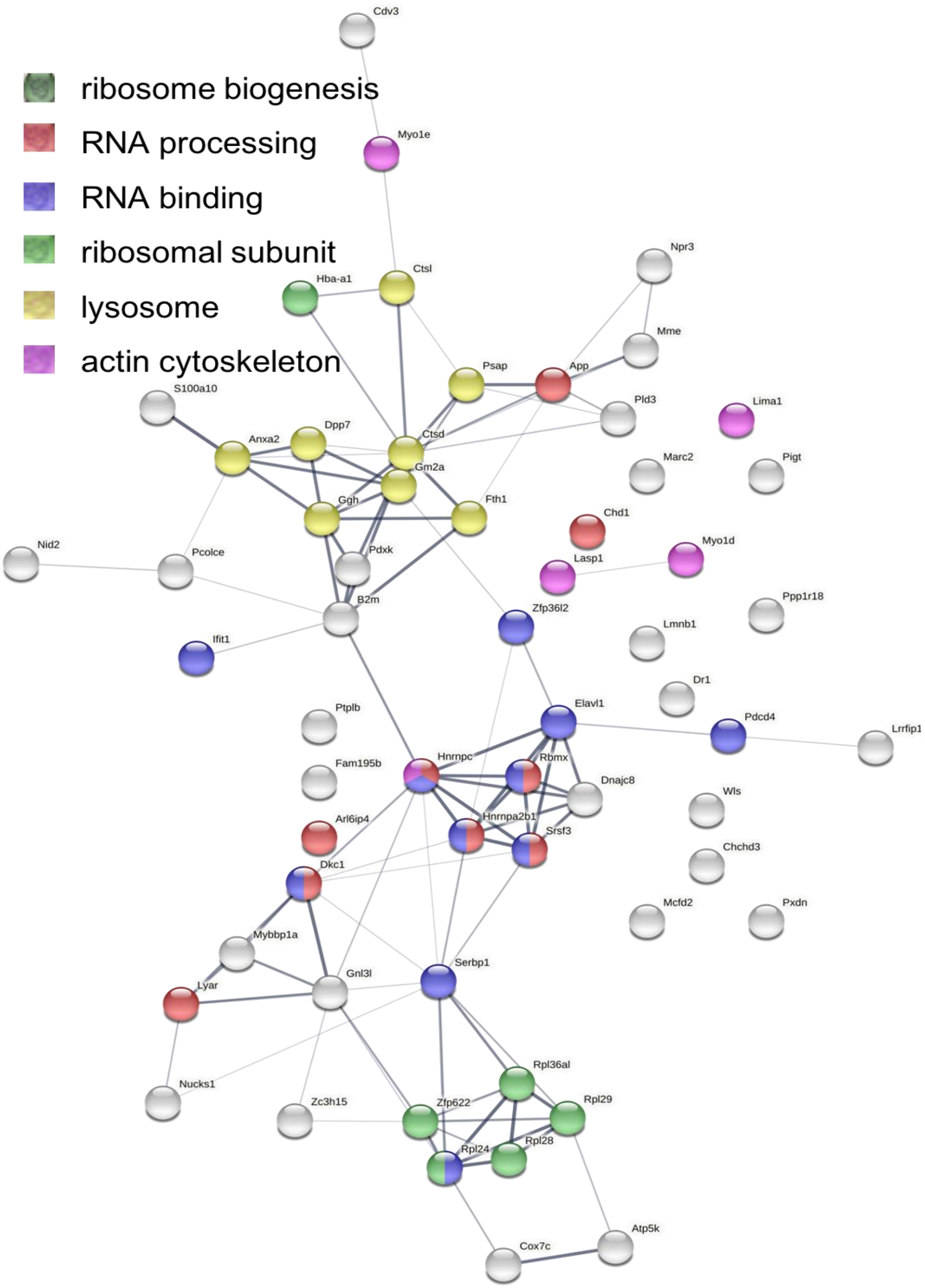

Supplementary Figure S2. Network analysis of 61 commonly regulated proteins by both H_2O_2 and Aß in 661 photoreceptor cells. Six colors separately indicate proteins in three groups including biological process, molecular function, and cellular components after GO enrichment analysis.
